# Unilateral, Small, Benign, Late-Onset, Large-Cell Calcifying Sertoli Cell Tumor: A Case Report

**DOI:** 10.7759/cureus.41614

**Published:** 2023-07-09

**Authors:** Markus Angerer, Christian Wülfing, Raphael Gübitz, Alexander Harms, Klaus-Peter Dieckmann

**Affiliations:** 1 Department of Urology, Asklepios Klinik Altona, Hamburg, DEU; 2 Department of Radiology, Asklepios Klinik Altona, Hamburg, DEU; 3 Institute of Pathology, MVZ Hanse Histologikum, Hamburg, DEU

**Keywords:** large cell calcifying sertoli cell tumor, testis-sparing surgery, germ cell cancer, sertoli cell tumor, testicular cancer

## Abstract

Large-cell calcifying Sertoli cell tumor (LCCST) is a rare, testicular sex cord, gonadal stromal tumor that belongs to the histological subgroup of Sertoli cell tumors. LCCSTs may involve malignant potential. However, metastasis is a rare phenomenon. We describe a case of benign late-onset LCCST with testis-sparing surgery. Modern imaging techniques were useful for considering organ-sparing surgery.

The ultrasound of a 37-year-old man disclosed a sharp demarcated and strong hyper-echoic lesion sized 1.5 cm, with broad dorsal acoustic shadowing. Testicular tumor markers, including lactate dehydrogenase (LDH), alpha-fetoprotein (AFP), and Beta-human chorionic gonadotropin (ß-HCG) did not reveal any pathological finding. Contrast-enhanced MRI of the pelvis showed a ring-shaped tumor with a strong contrast medium enhancement. Sections of the tumor showed a hard mass with a white calcified ring. A frozen section examination of the testicular tumor did not indicate malignancy. Histologic examination revealed a prominent and noticeable calcification of approximately 3 mm thickness. Tumor cells presented in the form of solid nests, tubules, and cords.

Our present case differs from previously reported LCCST cases because the tumor was unilateral, smaller in size, and presented in an older patient.

## Introduction

Testicular germ cell tumor (GCT) is a rare disease, accounting for no more than 1.5% of all neoplasms in males but representing the most common tumors in adolescents and young men in Western countries [1]. Only 5% of all testicular tumors belong to the subgroup of sex cord gonadal stromal tumors (SCSTs), which are mostly of benign nature, but less than 10% follow a malignant course [2].

Radical inguinal orchiectomy is the standard treatment for malignant testicular neoplasms. However, benign tumors can safely be managed with testis-sparing surgery (TSS). A thorough preoperative evaluation of a testicular mass is thus mandatory for considering conservative surgery. Small lesion size is a characteristic feature of benign tumors. A systematic review by Henriques et al. on small testicular masses showed the percentage of benign tumors to be 58.3% overall, 55.8% and 68.8% in testicular masses < 2.5 cm, and <1 cm, respectively [3].

High-frequency (>10 MHz) testicular ultrasound (US) is the current diagnostic standard for evaluating testicular lesions. To date, surgery is usually only performed after sonographic confirmation of a clinically suspected mass. Testicular tumors typically appear as hypo-echoic lesions of any size, with signals of color-coded duplex sonography in the corresponding lesion. Benign lesions are also hypo-echoic but with sharply demarcated margins on US [4].

Based on constantly improving US technology, an increasing number of small and non-palpable testicular tumors have been detected in recent years. Many of these incidentally detected lesions are of benign nature, but quite a number receive over-treatment with radical orchiectomy [3].

Calcifications on US are rare, in both benign and malignant lesions. Calcified testicular masses may represent benign spermatic granuloma, trauma, tuberculosis, filariasis, calcified Leydig cell tumor, as well as malignant embryonal carcinomas or teratomas [5].

Magnetic resonance imaging (MRI) of the scrotum with gadolinium-based contrast medium application provides higher sensitivity and specificity than scrotal US in the diagnosis of testicular masses and should be considered in cases remaining inconclusive after US [4]. 

Here, we report the clinicopathological and imaging features of a patient with benign large-cell calcifying Sertoli cell tumor (LCCST).

## Case presentation

A 37-year-old healthy man of Caucasian descent presented with the wish for a vasectomy. His history was uneventful, with no previous genital diseases. The physical examination did not reveal any pathological findings; in particular, there was no gynecomastia.

**Figure 1:**
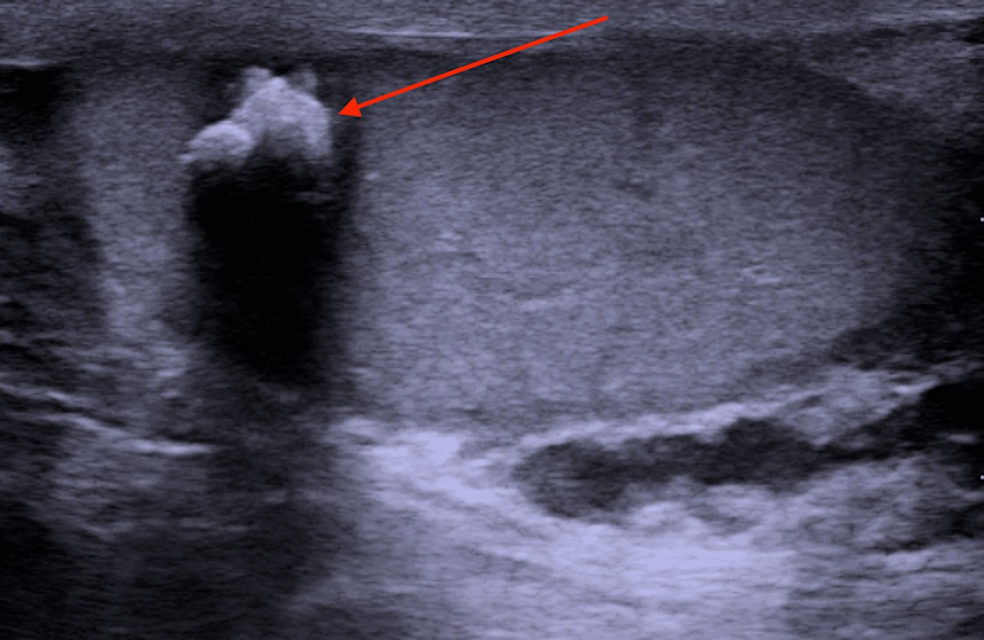
Ultrasound of the right testis Ultrasound image showing an 8 mm, right testicular calcified lesion (marked with the arrow)

Routine scrotal sonography disclosed a sharp demarcated and strong hyper-echoic lesion, sized 1.5 cm at the upper pole of the right testis, with broad dorsal acoustic shadowing (Figure 1). No signals of color-coded duplex sonography could be detected within the lesion. The left testis was normal upon physical examination and US.

Blood sample analysis did not reveal any pathological findings. In particular, serum tumor markers alpha-fetoprotein (AFP), beta-human chorionic gonadotropin (ß-HCG), and lactate dehydrogenase (LDH) were within normal limits. Likewise, serum testosterone, serum estrogen levels, luteinizing hormone (LH), follicle-stimulating hormone (FSH), prolactin, and sexual hormone-binding globulin (SHBG) were within normal limits.

**Figure 2:**
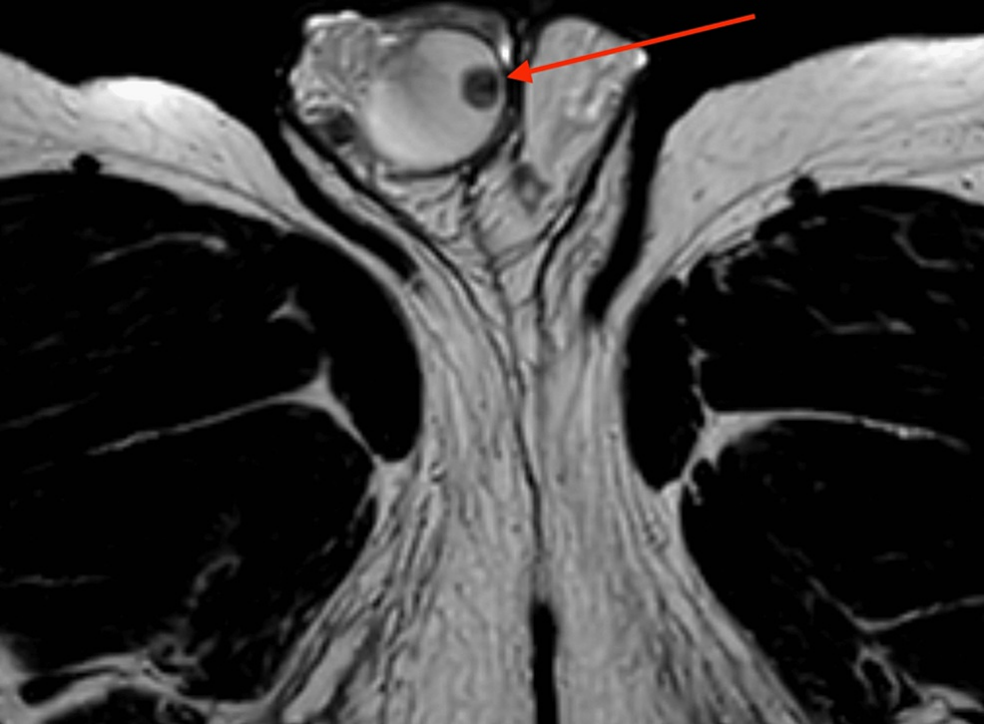
T2-weighted transversal MRI of the pelvis Image of the scrotum and right testicle and transversal view of both thighs showing an inhomogeneous, hypo-intense mass with a smooth border in the right testicle (marked with the arrow)

**Figure 3:**
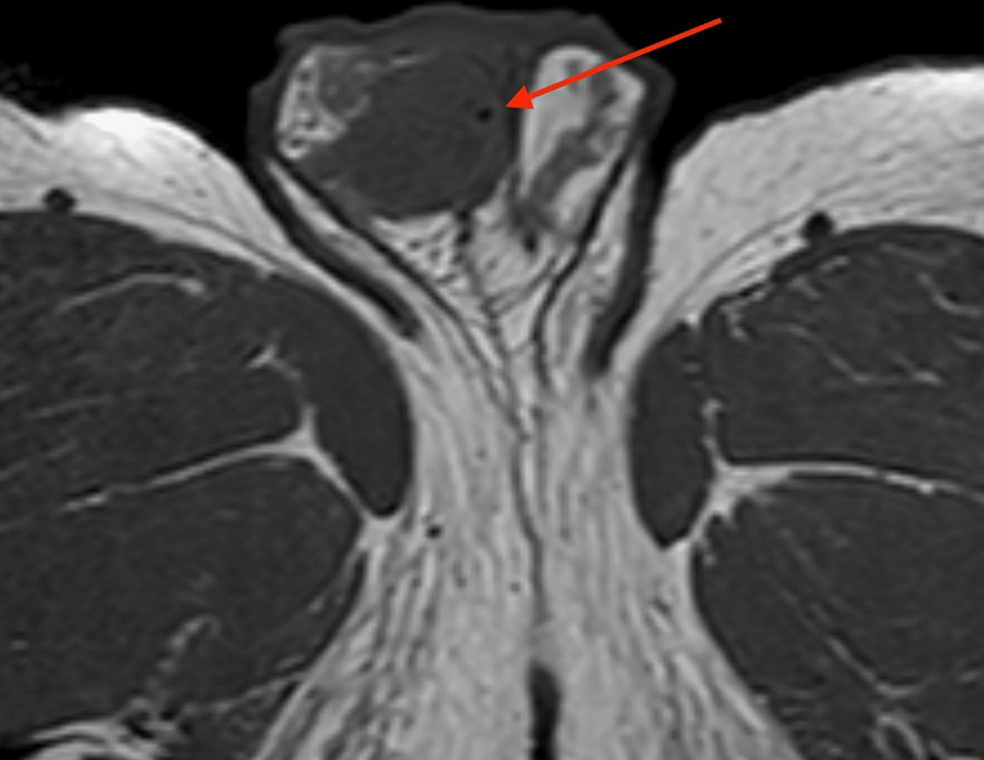
T1-weighted transversal MRI of the pelvis Image of the scrotum and right testicle and transversal view of both thighs showing a punctiform signal gap in the tumor center in the right testicle (marked with the arrow)

**Figure 4:**
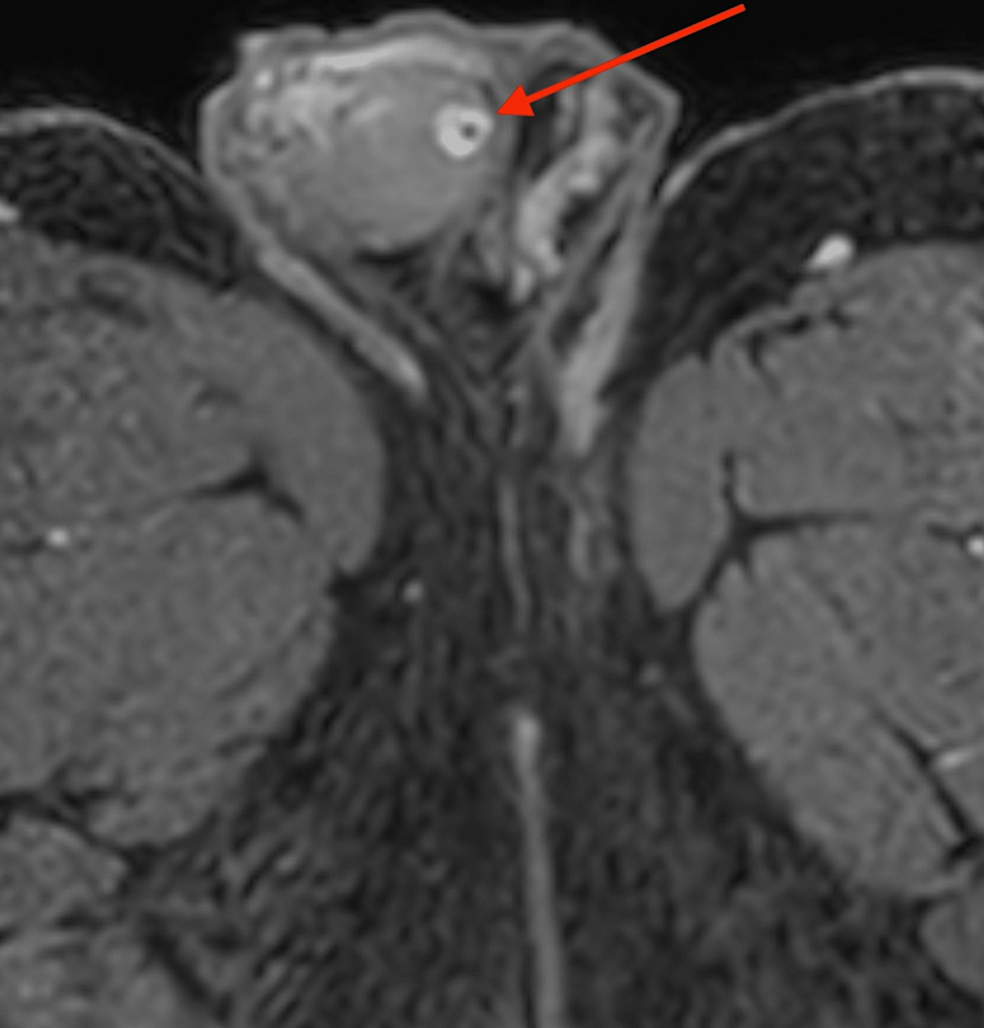
Contrast-enhanced, fat-suppressed T1-weighted transversal MRI of the pelvis Image of the scrotum and right testicle and transversal view of both thighs showing a ring-shaped, strong enhancement with contrast medium sparing of the hypo-intense tumor center in the right testicle (marked with the arrow)

**Figure 5:**
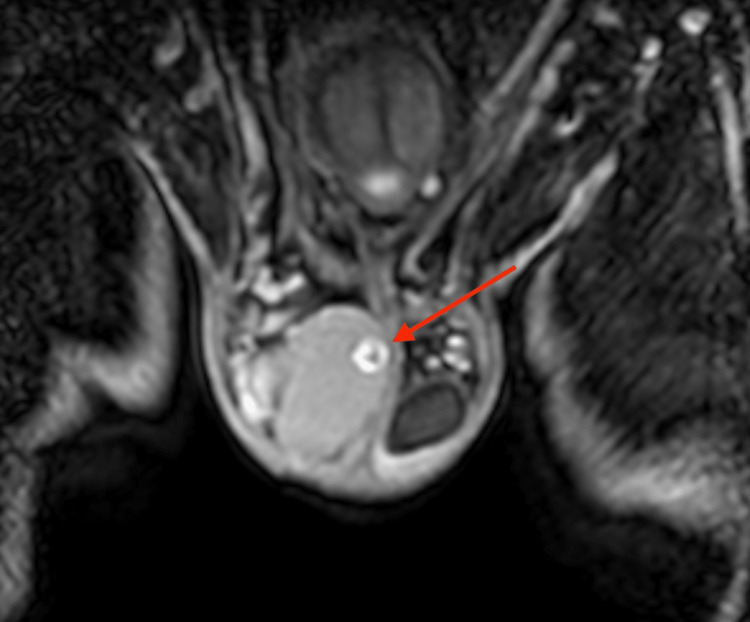
Contrast-enhanced, fat-suppressed, T1-weighted coronal MRI of the pelvis Image of the scrotum and right testicle and coronary view of both thighs showing a contrast-enhanced tumor in the right testicle (marked with the arrow)

An oval-shaped, 8 mm mass underneath the tunica albuginea testis was detected by additional magnetic resonance imaging using a 1,5 Tesla MRI with a surface coil. T2-weighted images revealed an inhomogeneous, hypo-intense mass with a smooth border (Figure 2). In pre-contrast T1W, there was a punctiform signal gap in the center of the lesion (Figure 3). Contrast-enhanced, fat-suppressed T1W imaging showed a ring-shaped strong enhancement with contrast medium sparing of the hypo-intense tumor center (Figures 4, 5). Furthermore, MRI showed a predominantly signal omission in diffusion-weighted imaging (DWI). The right paratesticular structures were normal upon MR imaging while a 2.4 cm spermatocele was detected at the caput of the left epididymis.

**Figure 6:**
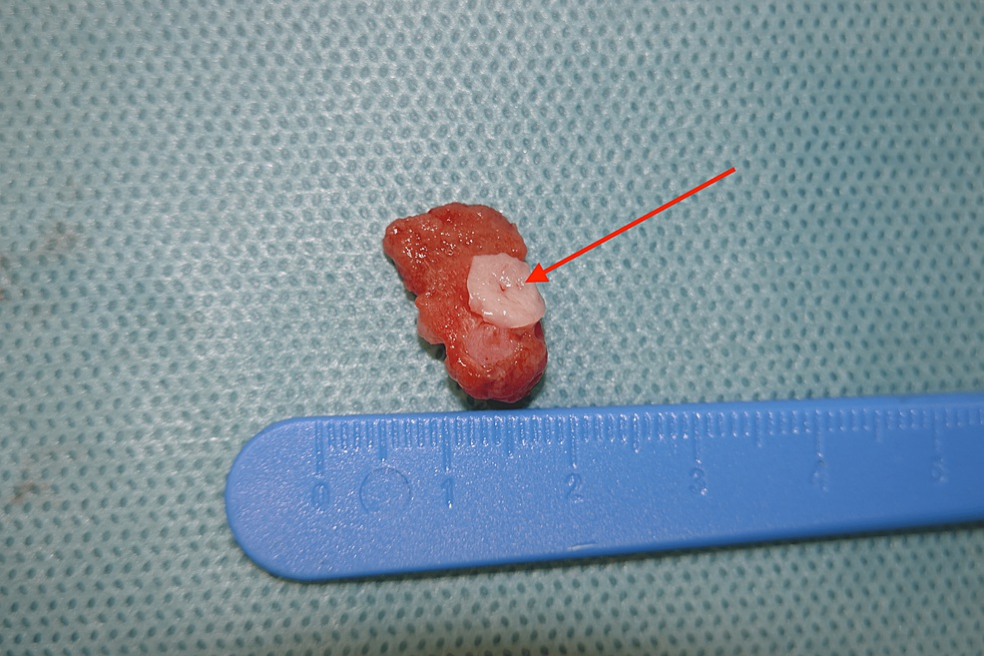
Macroscopic image of the tumor Sagittal section of the tumor shows a white calcified ring (marked with the arrow) and surrounding testicular tissue

During trans-scrotal exposure, the hard nodule was excised from the upper pole region, with preservation of the right testis. Gross pathologic examination during surgery revealed a hard mass with a white calcified ring at the excisional margin and brownish soft tissue in the center of the lesion (Figure 6). Frozen section examination did not indicate malignancy. Therefore, only two parenchymal biopsies adjacent to the excisional margin were performed additionally but no orchiectomy. The surgery and postoperative course were uneventful. Computed tomography of the abdomen did not reveal any pathologic findings. The patient is well one year after surgery.

**Figure 7:**
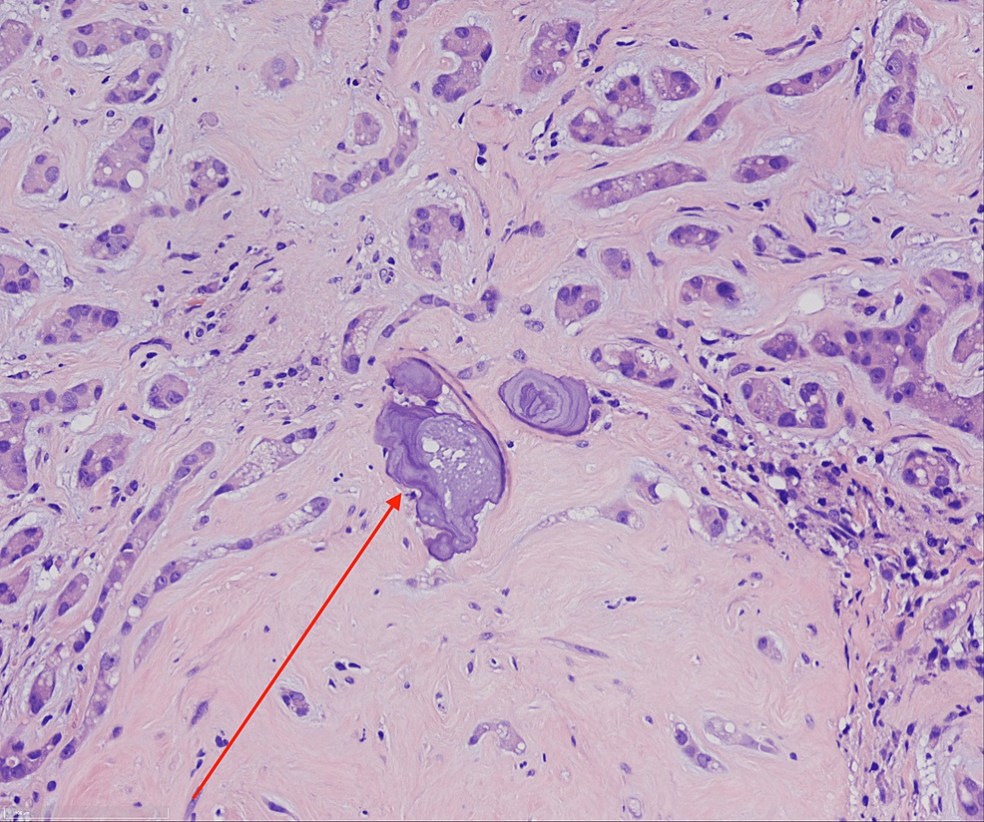
LCCST: psammomatous calcification (H&E stain; x20) LCCST = large-cell calcifying Sertoli cell tumor

**Figure 8:**
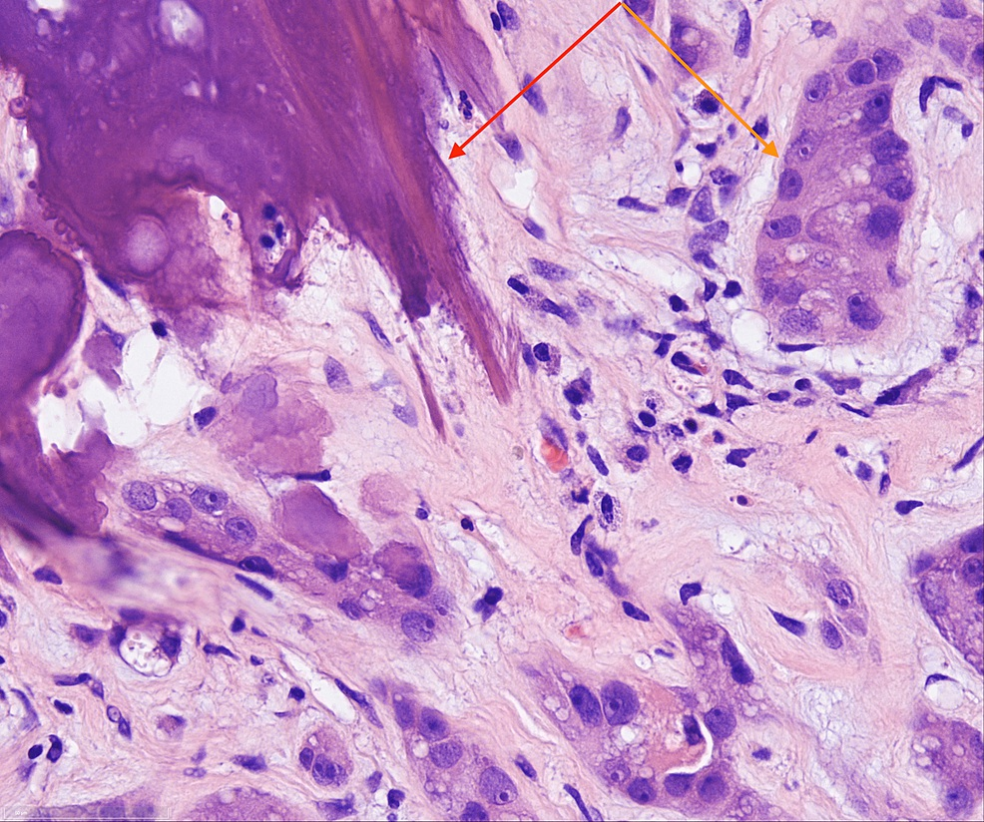
LCCST: tumor cells with ossification (marked with red arrow) Tumor cells exhibit cords and nests in the myxoid stroma with neutrophilic infiltrate (marked with orange arrow) (H&E stain; x40) LCCST = large-cell calcifying Sertoli cell tumor

**Figure 9:**
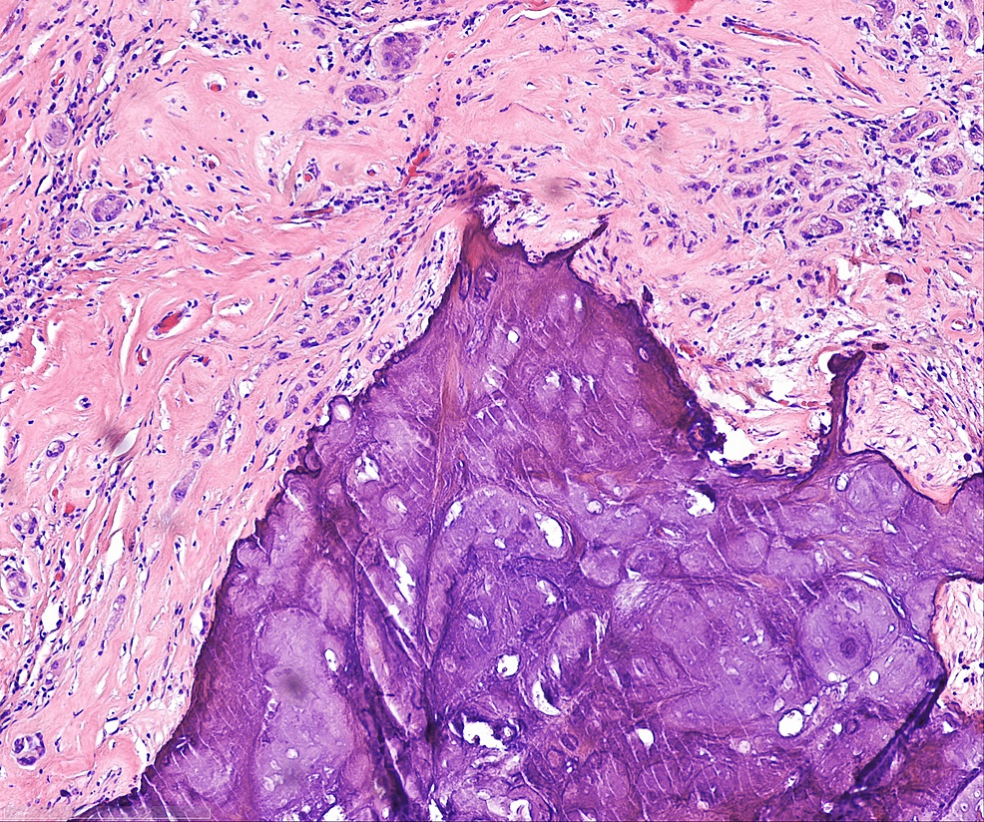
LCCST: prominent macrocalcification (H&E stain; x10) LCCST = large-cell calcifying Sertoli cell tumor

Histologic examination revealed an epithelioid neoplasm approximately 7 mm in size. Tumor cells are presented in the form of solid nests, tubules, and cords. Moreover, tumor cells were uniformly configured with strong eosinophilic cytoplasm and medium-sized cell nuclei. Most cells had prominent nucleoli in their nuclear chromatin, as well as vehicular-shaped nucleoli. The tumor was composed of small tubular structures with pale basophilic materials (Figures 7, 8). Calcification was prominent, with approximately 3 mm thickness (Figure 9). In the center of the neoplasm was a lymphocytic infiltration as well as fibrosis. The surrounding testicular parenchyma showed normal spermatogenesis.

Upon immunohistochemistry, the tumor cells were strongly positive for calretinin, S-100 protein, vimentin, inhibin, and Melan A, but negative for OCT4. Ki-67 index was 2%. There was no mitosis, necrosis, or vascular invasion.

In conclusion, the final diagnosis of a benign LCCST was made.

## Discussion

LCCST is a rare testicular SCST that belongs to the histological subgroup of Sertoli cell tumors (SCT). This neoplasm affects young patients in the majority of cases [6]. LCCSTs are morphologically characterized by the presence of massive calcification, and this feature was the leading sonographic symptom in the present case.

**Figure 10:**
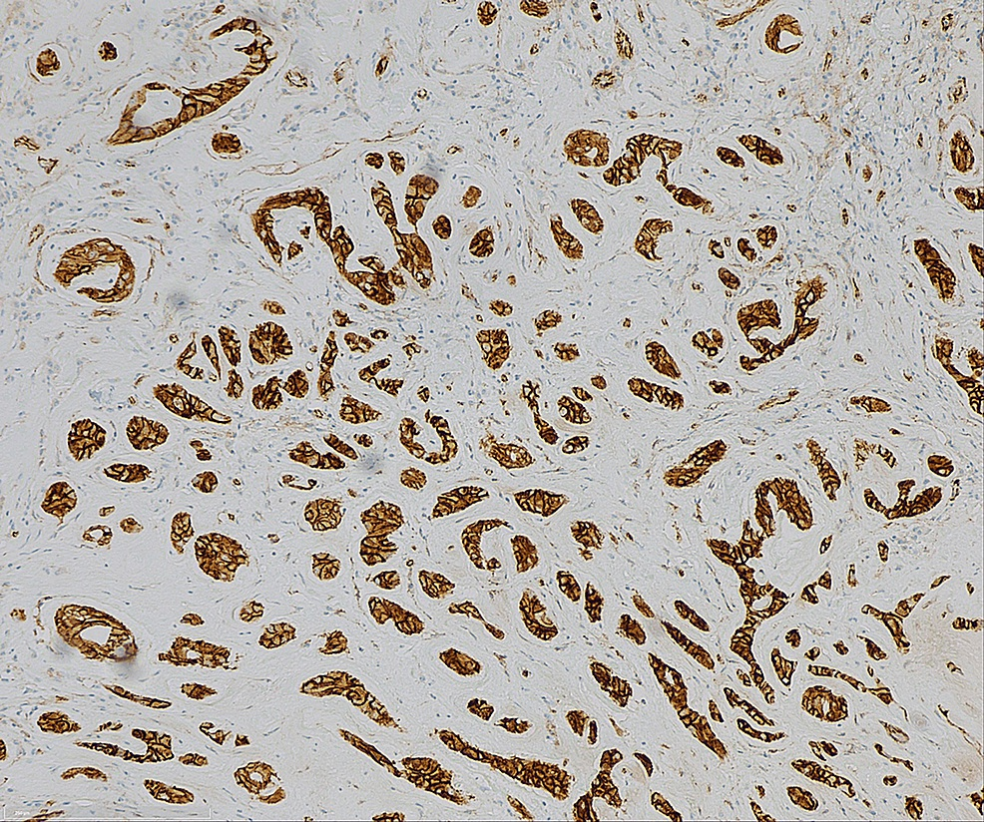
LCCST: cells show only characteristic cytoplasmatic nuclear β-catenin staining (x10) LCCST = large-cell calcifying Sertoli cell tumor

One of the reasons for separating LCCST as a distinct subtype from other SCTs in the “World Health Organization Classification of Tumors of the Urinary System and Male Genital Organs” is its different clinical presentation and histopathologic characteristics. This is based on the occurrence of CTNNB1 gene mutations and nuclear-catenin staining (Figure 10) [2,7].

Proppe and Scully provided the first description of LCCST cases in 1980 [8]. In 1994, Gierke et al. published the first sonographic images of an LCCST featuring a dense calcification and acoustic shadow [9]. The case reported herein presented very similar findings. A comprehensive review of the literature by Al-Obaidy et al. reported 97 cases of LCCST until 2022 [10].

SCSTs encompass 4% of all testicular tumors, and SCT comprises 1%, respectively [6]. Other SCSTs include SCT not otherwise specified, intratubular large cell hyalinizing Sertoli cell tumor, sclerosing Sertoli cell tumor, Leydig cell tumor, and granulosa cell tumor [6].

LCCST malignancy is a rare phenomenon that is characterized by two criteria, size >4 cm, extra-testicular growth, necrosis, vascular space invasion, and high mitotic index [11]. Malignant tumors are usually unilateral and solitary [12]. Up to date, only 18 cases of malignant LCCSTs have been reported [12].

Giglio et al. divided LCCSTs into two clinical subgroups, early-onset and late-onset LCCST [13]. Early-onset LCCSTs appear in the first two age decades and are commonly related to genetic syndromes such as the Carney complex and Peutz-Jeghers syndrome. Furthermore, early-onset LCCSTs seem to have benign characteristics. Late-onset LCCSTs (mean age is 39 years) are not associated with genetic disorders but may involve malignant potential [12-14]. LCCSTs are frequently presented multicentrically and/or bilaterally [12].

A large clinicopathological study revealed that median patient age and size were 15.5 years and 1.9 cm, 19 years and 1.6 cm, and 28.5 years and 2.3 cm, for benign, ambiguous, and malignant tumors, respectively [10].

Our case represents a typically late-onset LCCST that is associated with malignancy in many cases. However, malignant behavior was neither documented histologically nor clinically. In particular, the histologic findings of tumor size 7 mm and Ki-67 index of 2% are fully consistent with benign nature. We report a case of unilateral benign late-onset LCCST in a 37-year-old patient without any genetic disorders. LCCST is histopathologically associated with the presence of massive calcification; tumor cells have abundant eosinophilic cytoplasm and prominent nucleoli. 

There is a unique sonographic feature of the neoplasm, for example, hyper-echogenicity with acoustic shadowing, due to the calcified tissue. A differential diagnosis of malignant testicular calcified masses, such as teratomas, has to be made. However, calcifying non-seminomatous germ cell tumors are usually larger and likely heterogeneous in echogenicity with calcification [15].

Scrotal sonography in our patient clearly showed a round calcified lesion with acoustic shadowing, as well as peripheral vascularization. This unique feature was not consistent with malignant germ cell tumors and thus prompted considering TSS, as also advocated by the current German clinical practice guideline for the management of germ cell tumors [16]. Further support for the assumption of a benign neoplasm came from contrast-enhanced MRI imaging showing a ring-shaped strong enhancement. Al-Obaidy et al. suggest LCCSTs in patients above 25 years old should be considered potentially malignant, therefore inguinal surgical approach should be done [10].

Our present case is consistent with previously reported LCCST cases, with respect to histopathological features and ultrasonographic appearance. Minor differences to previous cases relate to unilateral presentation, smaller size, and older patients’ age. MR imaging of LCCST has been reported only exceptionally to date but this imaging technique proved useful in the present case to consider organ-sparing surgery.

## Conclusions

SCTs encompass around 1% of all testicular neoplasms. LCCSTs represent one rare subtype of SCTs that are further subclassified into early-onset and late-onset LCCSTs, with the latter usually representing malignant potential. Radical inguinal orchiectomy is the gold standard for malignant LCCSTs while organ-sparing surgery with intraoperative frozen section may be a good option for patients with benign LCCSTs. Preservation of remaining active testicular parenchyma may allow for maintaining endocrine function and fertility. The unique feature of LCCST is a circumscribed hyper-echoic lesion with acoustic shadowing upon ultrasonography.
